# Modeling tumor immunity of mouse glioblastoma by exhausted CD8^+^ T cells

**DOI:** 10.1038/s41598-017-18540-2

**Published:** 2018-01-09

**Authors:** Hiroshi Nakashima, Quazim A. Alayo, Pablo Penaloza-MacMaster, Gordon J. Freeman, Vijay K. Kuchroo, David A. Reardon, Soledad Fernandez, Michael Caligiuri, E. Antonio Chiocca

**Affiliations:** 1 Harvey W. Cushing Neuro-oncology Laboratories (HCNL), Department of Neurosurgery, Harvard Medical School and Brigham and Women’s Hospital, Boston, MA 02115 USA; 2 Department of Microbiology-Immunology, Feinberg School of Medicine, Northwestern University, Chicago, IL 60611 USA; 3 Department of Medical Oncology, Dana-Farber Cancer Institute, and Brigham and Women’s Hospital, Boston, MA 02115 USA; 4 Evergrande Center for Immunologic Diseases, Harvard Medical School and Brigham and Women’s Hospital, Boston, MA 02115 USA; 5 Center for Neuro-Oncology, Dana-Farber Cancer Institute, and Brigham and Women’s Hospital, Boston, MA 02115 USA; 6 Center for Biostatistics, The Ohio State University, Columbus, Ohio, 43210 USA; 7 Comprehensive Cancer Center, and Division of Hematology in Department of Internal Medicine, College of Medicine, The Ohio State University, Columbus, Ohio, 43210 USA

## Abstract

T cell exhaustion occurs during chronic infection and cancers. Programmed cell death protein-1 (PD-1) is a major inhibitory checkpoint receptor involved in T cell exhaustion. Blocking antibodies (Abs) against PD-1 or its ligand, PD-L1, have been shown to reverse T cell exhaustion during chronic infection and cancers, leading to improved control of persistent antigen. However, modeling tumor-specific T cell responses in mouse has been difficult due to the lack of reagents to detect and phenotype tumor-specific immune responses. We developed a novel mouse glioma model expressing a viral epitope derived from lymphocytic choriomeningitis virus (LCMV), which allowed monitoring of tumor-specific CD8 T-cell responses. These CD8 T cells express high levels of PD-1 and are unable to reject tumors, but this can be reversed by anti-PD-1 treatment. These results suggest the efficacy of PD-1 blockade as a treatment for glioblastoma, an aggressive tumor that results in a uniformly lethal outcome. Importantly, this new syngeneic tumor model may also provide further opportunities to characterize anti-tumor T cell exhaustion and develop novel cancer immunotherapies.

## Introduction

Malignant glioma and glioblastoma (GBM) are some of the most common primary central nervous system (CNS) tumors characterized by the accumulation of multiple genetic mutations resulting in uncontrollable cell proliferation and tumor heterogeneity^1^. GBM phenotypes include tumor neovasculature, necrosis, invasion, and immunosuppression. Despite advances in surgery, chemotherapy, and radiotherapy, these cancers result in a uniform mortality, with survival at five years from diagnosis being exceedingly rare^1^. Recent advances in the understanding of how immune responses are regulated during chronic viral infection have led to the discovery of several inhibitory pathways that regulate CD8 T cell effector functions^2–5^, and this has translated into novel treatments for solid tumors. Although the central nervous system (CNS) was once thought to be an immune privileged organ, it is now evident that immune cells can infiltrate the CNS to control pathogens and cancers^6–8^. Several immunotherapeutic approaches are being tested for the treatment of GBMs, including CAR-T cells, peptide/nucleic acid vaccination, immune checkpoint blockade, oncolytic and gene therapy, monoclonal antibodies targeting co-stimulatory pathways, and adoptive cell therapies^9–12^.

Adaptive immunity against tumors depends on cytotoxic CD8^+^ T lymphocytes (CTLs) to control and eliminate cancer cells in a durable manner. However, when CTLs infiltrate into the tumor, several immunosuppressive pathways may hinder tumor rejection^13–15^. For example, T cell activation becomes inhibited by immune checkpoint signaling pathways, such as PD-1/PDL-1 and others^2,16,17^, leading to T-cell exhaustion^2–5^. Recent studies suggest that these signaling pathways contribute to GBM^18–22^ and clinical trials are testing the ability of immune checkpoint inhibitors to treat these aggressive cancers. Seminal studies in the mouse model of chronic LCMV infection revealed that treatment with PD-1 (or PD-L1) blocking antibodies results in significant improvement in T cell function and enhanced antiviral control^2^. These initial findings were soon generalized to various tumor models in mice, and currently PD-1 blockade constitutes a treatment for many types of cancers in humans. This is thought to be also relevant for aggressive cancers, including GBM where CD8 T cells could become dysfunctional due to chronic exposure to tumor antigens^9,23–26^. Several transplantable and genetically engineered preclinical mouse models of GBM exist, where tumors have a relatively limited time frame to grow and establish. This limited time frame may render difficult the chronic presentation of tumor antigens, necessary to induce the “exhaustion” of CTLs. This may potentially magnify the success of tested immunotherapies without recognizing the “true” state of immunocompromise^27^. We thus hypothesized that a previously published model of CTL “exhaustion” that occurs in mice during a chronic LCMV infection could be adapted to recapitulate this dysfunctional state of anti-tumor T-cell immunity in mice models of GBM^2,3,28,29^.

Here we show that mice chronically infected with a chronic LCMV strain (Clone 13; Cl13), which induces T cell exhaustion, are unable to reject orthotopic, syngeneic mouse gliomas that express the LCMV GP33 epitope. In contrast, mice infected with an LCMV strain (Arm) that leads to an acute, self-limited infection that induces functional T cell memory, efficiently reject the same GP33 epitope -expressing glioma cells. The failure to reject mouse glioma tumors correlates with high expression of PD-1 in CTLs of Cl-13 mice. Interestingly, this inability to reject mouse gliomas can be partially reversed by treatment with an antibody against PD-1. Altogether, we develop a novel mouse model of cancer that can be used to model the exhausted state of CTLs in GBM and other cancers, and that can be used to evaluate and discover effective immunotherapies.

## Results

### Failure to reject glioma cells that express the LCMV GP33 epitope in mice chronically infected with LCMV Cl-13

**Figure 1:**
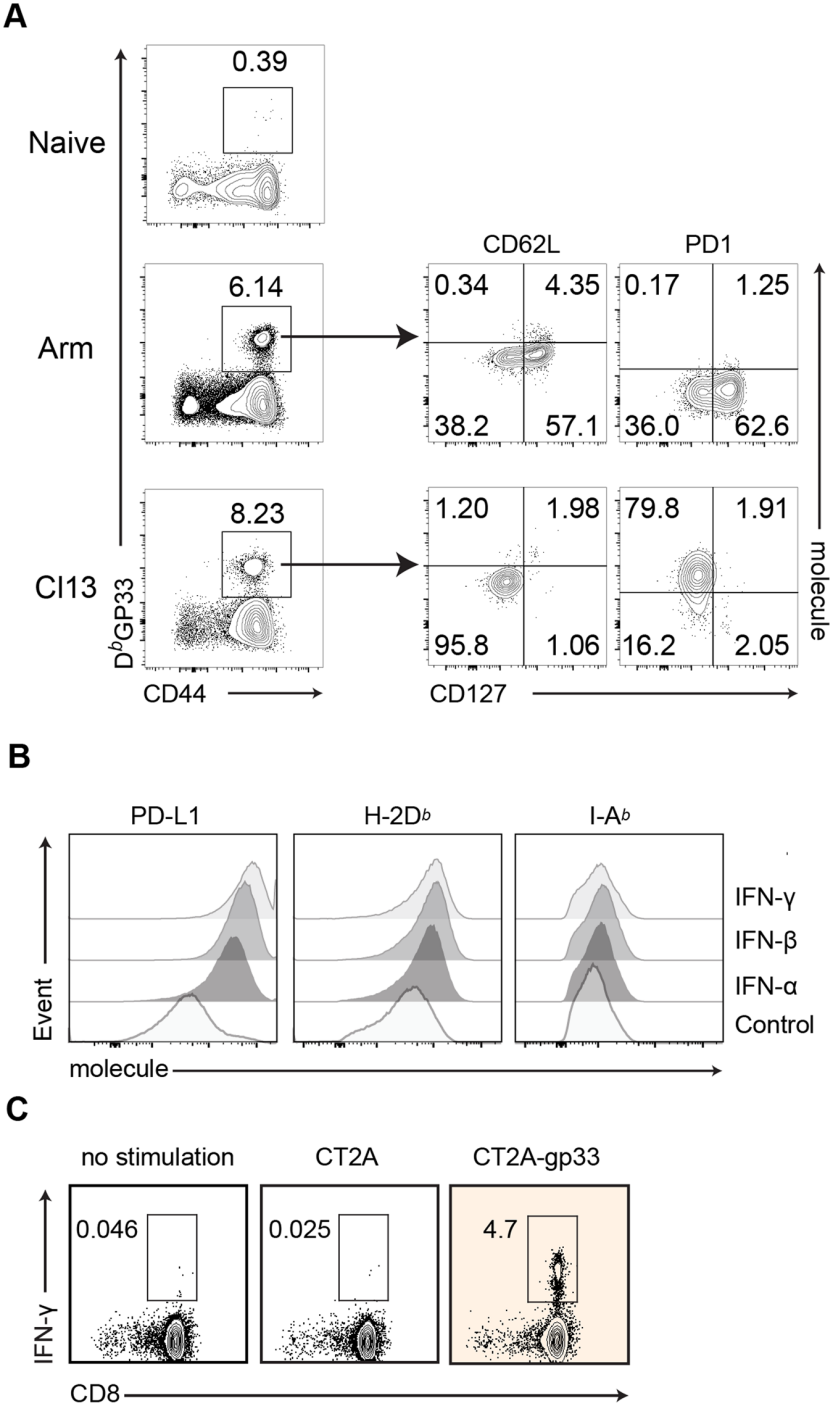
Generation of LCMV GP33 epitope expressing CT2A glioma line and Cl13-induced PD-1^hi^GP33^+^CD8^+^ T cells. (**A**) Phenotypic analysis of GP33-specific CD8^+^ T cells in PBMCs of B6 mice infected with LCMV Arm or Cl13, 20 days after infection and before tumor challenge (see Fig. 2 for schematic). The plots are gated on Live^+^, CD3^+^ and CD8^+^ cells. Molecule on Y-axis *(right subplots)* denotes CD62L or PD-1(labeled top of subplots). Similar results were obtained from mice used in Fig. 2A. (**B**) Histograms showing the frequency of PD-L1, MHC Class I (H-2D^b^) and II (I-A^b^) molecules expression on clonal CT2A-gp33 cell populations after exposure to IFN-α, -β (each 500 units/mL) and -γ (20 µg/mL) (**C**) FACS plots showing the frequencies of intracellular IFN-γ expressing CD8^+^ lymphocytes, which were isolated from the spleen of LCMV Arm infected mice after 5 hours of no co-culture (*left*) or co-culture with CT2A (*center*) or CT2A-gp33 (*right*).

We hypothesized that sharing the same antigen between a tumor and a chronically infectious virus would permit the establishment of a preclinical mouse model that would mimic dysfunctional T-cell immunity. Such a model may be used to study the impact of pre-existent, yet dysfunctional or “exhausted”, host immunity in cancer immunotherapy. The previously published LCMV infection mouse model takes advantage of two different strains of LCMV^2,28^. LCMV Arm sets up an acute infection in mice that is cleared resulting in functional memory CD8^+^ T cells. On the other hand, LCMV Cl-13 sets up a chronic infection that generates GP33^+^ CD8^+^ T cells that express high levels of PD-1 and are functionally exhausted, as early as 9 days after infection^30,31^. At day 20 post-infection, LCMV Arm and LCMV Cl-13 infected mice had approximately equal percentages of activated (CD44+) GP33-specific CD8 T cells (Fig. 1A, *left subplots*). However, these GP33-specific CTLs displayed different phenotypic markers in acute vs chronic infection. CD127^+^CD62L^+^ (central memory) and CD127^+^CD62L^−^ (effector memory) cells were PD-1^low^ in Arm (0.17%), but were rare and GP33^+^ effector cells display PD-1^high^ (79.8%) in Cl13-infected mice (Fig. 1A, *right subplots*, *left upper quadrants*). These findings agreed with those from previous publications^32,33^ showing that Arm infection generated highly functional memory CD8^+^ T cells, in contrast to Cl13 infection that induced an exhausted PD-1^high^ CD8^+^ T cell population.

We utilized mice infected with either LCMV Arm or Cl-13 to interrogate how they would respond to an intracerebral challenge with CT2A glioma that expresses the cognate LCMV antigen GP33 (designated CT2A-gp33). CT2A-gp33 glioma cells expressed increased levels of the class I major histocompatibility complex (MHC) molecule H-2D^*b*^ and of PD-L1, upon stimulation with IFN*α*, β, or *γ* (Fig. 1B). There was no change in the expression of the MHC class II molecule I-A^b^. We confirmed that CD8 T cells in LCMV Arm-immune mice recognized CT2A-gp33 cells (Fig. 1C).

**Figure 2:**
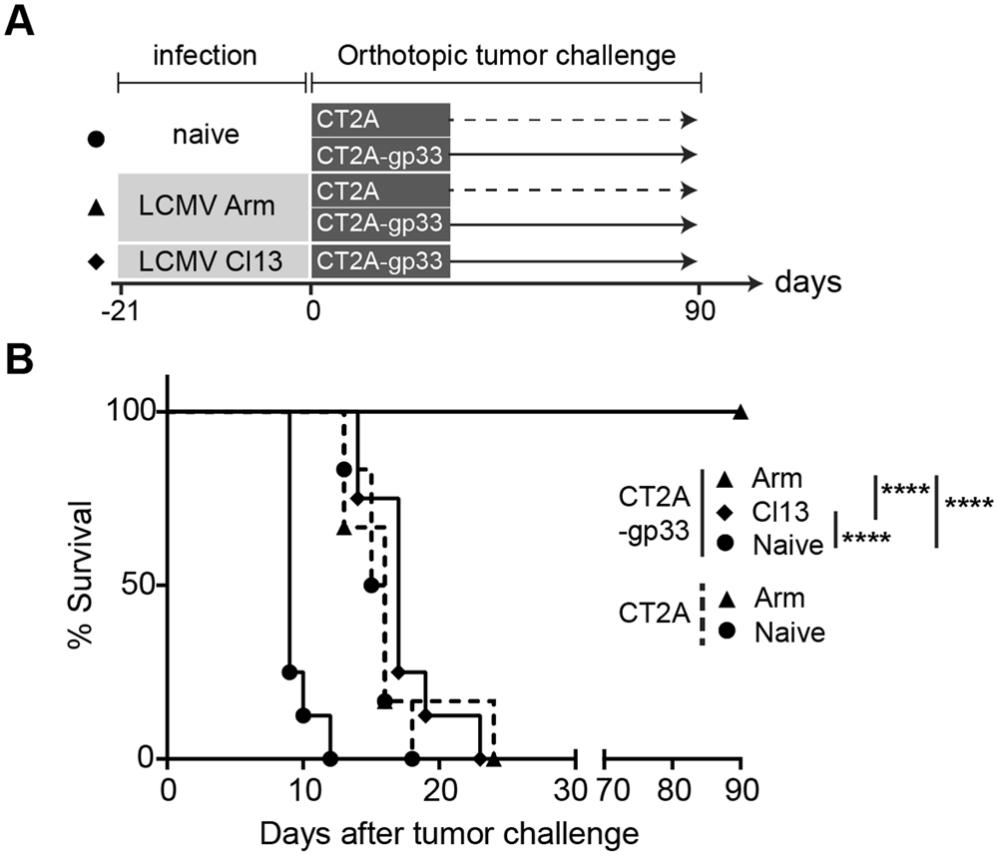
GP33 antigen-dependent rejection of orthotopic gliomas in LCMV Arm-infected, but not Cl13-infected mice. (**A**) Experimental set-up for the orthotopic glioma challenge in LCMV-infected or naive B6 mice. Twenty-one days before tumor challenge, LCMV Arm (acute; *triangle*) or Cl13 (chronic; *diamond*) strain were administered to B6 mice. Age-matched naive B6 mice (*circle*) were used as a comparison. Challenge were glioma cells: CT2A (100,000 cells; *dotted line*) or CT2A-gp33 (400,000 cells; *solid line*) which were implanted into the right brains stereotactically for the survival study (shown in *B*). (**B**) Survival analyses after orthotopic tumor challenge. Survival was monitored for up to 90 days. Median survival after CT2A challenge (n = 6 in each group) was 15 (naive) and 16 (Arm) days, while median survival after CT2A-gp33 challenge (n = 8 in each group) was 9 (naive), >90 (Arm) and 17 (Cl13) days. Log-rank test was used to compare survival among the LCMV infection (Arm, Cl13) and naïve in CT2A-gp33 challenge groups, or between Arm and Naïve in CT2A challenge groups, ****P < 0.0001.

Next, we characterized the survival of mice challenged with CT2A-gp33 or CT2A gliomas (Fig. 2A). All of the LCMV Arm-infected mice survived an intracerebral challenge with CT2a-gp33 glioma cells, while none of the LCMV Cl-13 infected mice did (Fig. 2B). As expected, no mice survived a challenge with parental CT2A glioma cells that did not express the cognate LCMV antigen. This indicated that PD-1^high^ expressing CTLs were not functionally effective against the CT2A presenting GP33 epitope in Cl-13 mice.

**Figure 3:**
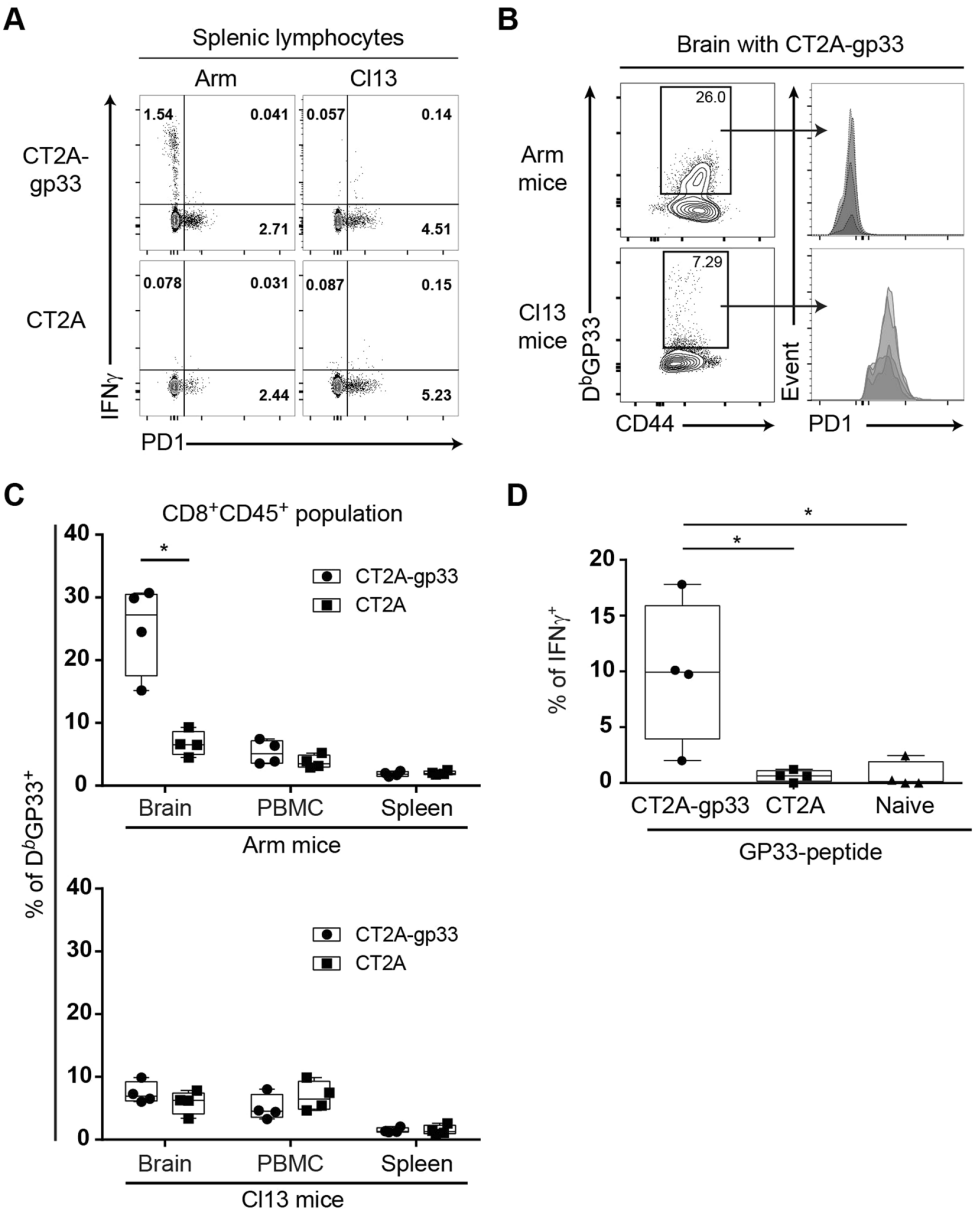
Frequency of GP33 antigen specific CD8+ T cells with PD-1 expression in the brains of Arm vs. Cl13 mice. (**A**) FACS plots showing the frequencies of intracellular IFN-γ and surface PD-1 expressing CD3^+^CD8^+^ T cells at five hours after *ex vivo* co-culture of splenic lymphocytes harvested from mice infected with LCMV Arm (*left*) or Cl13 (*right*), with CT2A-gp33 (*top*) or CT2A (*bottom*). Plot represents one of two replicates. (**B**) Histograms of PD-1 expression of GP33^+^lymphocytes (*square* in the *left* panel) after gating on Live^+^CD45^+^CD8^+^ T cells. Histograms (*right*) of four samples are overlapped while FACS plot (*left*) shows one; (**C**) Percentages of gp33-tetramer positive staining for CD8^+^CD45^+^ cells isolated from brains, PBMC or spleens of Arm- *(upper graph)* or Cl13-preinfected mice at seven days after orthotopic challenge with CT2A-gp33 (*circles)* or CT2A (*squares)*. GP33^+^lymphocytes are selected as shown in the square in the left panel B after gating on Live^+^CD45^+^CD8^+^ T cells. (**D**) Percentages of brain-infiltrating IFNγ -expressing CD8^+^ T cells, harvested from 7-day old CT2A-gp33 (*left)*, CT2A *(center)* or no-tumor control (right) challenged naive (*non LCMV-infected*) mice, 5 hours after *in vitro* incubation with GP33 peptide. Whiskers in box plots indicate maximum and minimum values measured (n = 4; each group), while line indicates the median. Statistical analyses by Student’s *t*-test, where *p < 0.05.

To characterize these contrasting responses against CT2A-gp33, we performed an *ex vivo* assay with splenocytes derived from Cl13 vs. Arm mice. There was an IFN-*γ* ^+^ PD-1^low^ population in CD8 T cells in Arm-immune mice in response to CT2A-gp33 (Fig. 3A). In Arm-immune mice, at day 7 of tumor challenge, there was a significant expansion in GP33^+^ CTLs among brain-infiltrating lymphocytes compared to Cl13 mice (Fig. 3B). PD-1 expression of these GP33^+^ CTLs in Cl13 mice was elevated when compared to that in Arm mice (Fig. 3B). CD44 expression on GP33+ CTLs was also down-regulated in Cl13 vs. Arm mice, although there was still some degree of expression in most of these cells (supplementary Figure 1). The observed GP33^+^ CTL expansion in Arm-immune mice was specific to the brain and was not observed in lymphocytes obtained from spleen or blood after CT2A-gp33 or CT2A tumor challenge (Fig. 3C). To provide evidence that CT2A-gp33 challenge directly elicits effector T cell development against the GP33 epitope in the brain, we challenged naïve (*i.e*., *not LCMV-infected)* mice. There was also a significant increase in the percentage of IFN*γ*-expressing T cells in response to the GP33 epitope (comparing CT2A-gp33 vs. CT2A) in brains from non- LCMV infected naïve mice, showing that the GP33 antigen could effectively prime brain-infiltrating CD8^+^ T cells against CT2A-gp33 gliomas in brain (Fig. 3D). Taken together, we developed a model that allows for the easy tracking, phenotypic characterization and tumor protection evaluation by tumor-specific T cells.

### PD-1 blockade rescues mice from CT2A-gp33 glioma challenge in the LCMV Cl13 model

**Figure 4:**
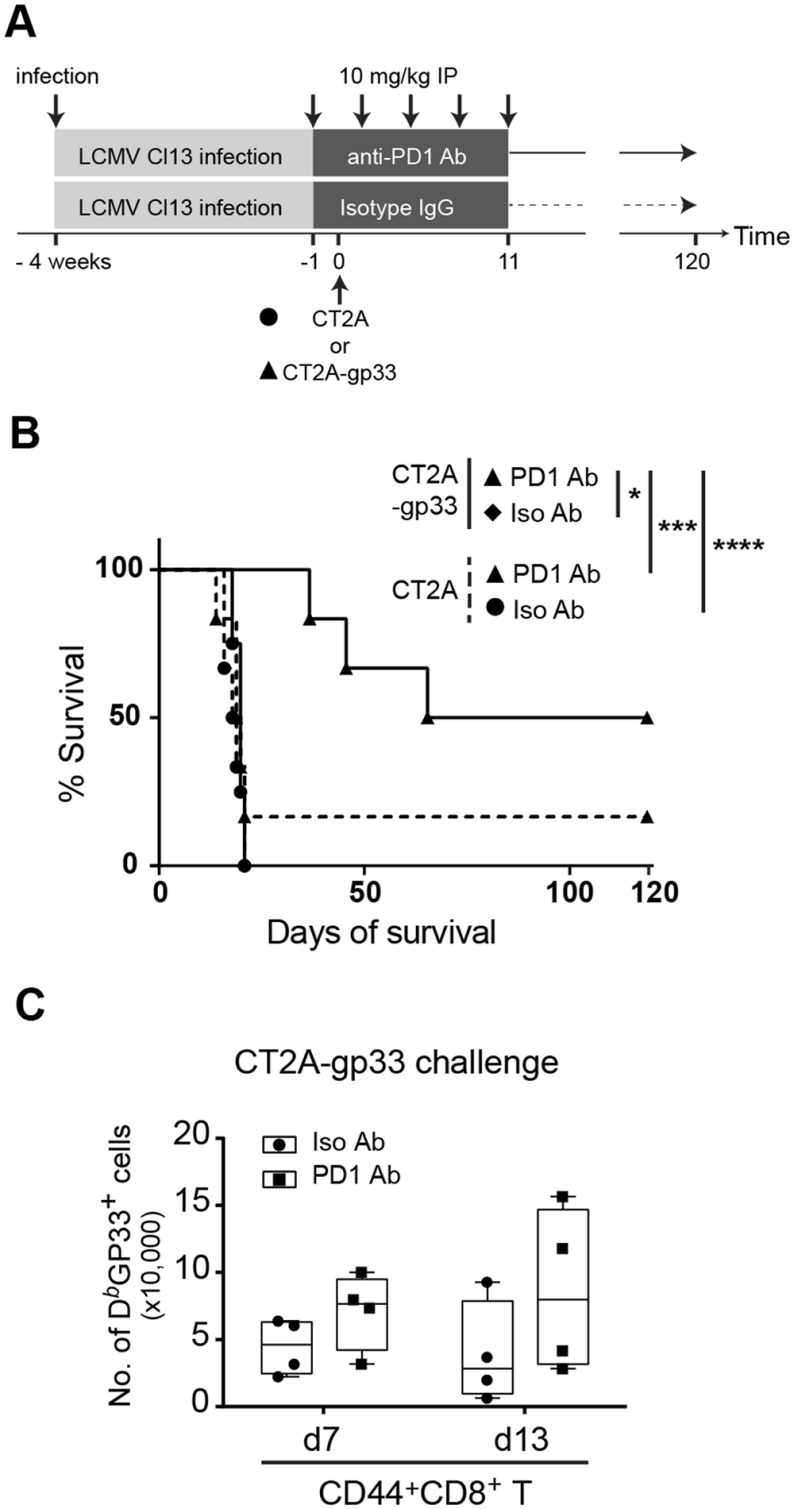
PD-1 blockade restores anti-glioma immunity in Cl13 mice. (**A**) Experimental set-up of the survival study showing the timing of orthotopic glioma challenge and intraperitoneal injections of therapeutic antibodies in LCMV Cl13 infected B6 mice. (**B**) Survival after orthotopic tumor challenge was monitored for up to 120 days. Median survival times after CT2A challenge with anti-PD-1 Ab or its isotype IgG were 20 (n = 4) and 18.5 (n = 6) days, respectively and those after CT2A-gp33 challenge with anti-PD-1 Ab or isotype IgG Ab treatment were 93 (n = 6) and 19.5 (n = 6) days, respectively. Log-rank test was used to compare survival between anti-PD-1 Ab vs isotype IgG in CT2A-gp33 vs CT2A groups, *p < 0.05, ***p < 0.001, ****p < 0.0001. (**C**) Numbers of GP33-specific effector CD44^+^ CD8^+^ T cells in CT2A-gp33-bearing brains of Cl13 pre-infected mice, 7 and 13 days’ post-tumor challenge, after treatment with PD-1 antibody or control isotype.

We next interrogated if PD-1 blockade can improve the control of CT2A-gp33 gliomas in mice infected chronically with LCMV Cl-13 (Fig. 4A). One day before tumor challenge, we administered a PD-1 blocking or a control antibody every third day for five times (Fig. 4A). PD-1 blockade resulted in a significant improvement in the survival of Cl13 pre-infected mice challenged with CT2A-gp33, when compared to control treated mice (Fig. 4B). PD-1 blockade was effective only when the GP33 epitope was expressed on CT2A cells. We next characterized PD-1 expression on GP33-specific CD8^+^ T lymphocytes in brains after PD-1 antibody treatment, at 7 and 13 days after tumor challenge. There was a trend for an increase in the numbers of GP33^+^CD8^+^ T cells in response to PD-1 blockade antibody compared to the isotype control (Fig. 4C). Taken together, these results thus showed that PD-1 blockade rescued the capacity of Cl13-immune mice to reject CT2A-gp33 glioma challenges with an increase in GP33^+^ CTLs. These findings show that PD-1 blockade could be a potent therapy to improve T cell function during both chronic infections and cancers.

## Discussion

Tumor recurrence is universal in GBM patients even after standard therapies such as gross total resection and chemoradiation. The failure of immune surveillance against residual tumor cells is a likely cause for this. One immune evasion mechanism includes the long-term exposure of tumor antigens causing anti-tumor immune cells into a state of progressive dysfunction or exhaustion. Several mouse models where tumors are implanted show evidence that tumor-infiltrating T lymphocytes possess an exhausted phenotype with expression of immune-checkpoint receptors and impaired anti-tumor function^34^. The same T cell phenotypes were initially reported in chronic viral infection using LCMV^2,4,19,33,35,36^. For GBM, a transplantable mouse model using mouse GL261 is commonly utilized, although this model has been criticized for its relatively high immunogenicity that amplifies the outcome of immunotherapy^9,37,38^. Here, we hypothesized that mice with pre-existent exhausted T cell immunity against a specific antigen would not be able to reject a challenge with GBM cells that expressed the same antigen. We show that: **a-** The previously reported model of acute (Arm) vs. chronic (Cl13) LCMV infection could be employed in modeling antigen-specific T cell exhaustion against cancer cells; **b-** This correlated with PD-1^high^ GP33^+^ T cells in the brains of Cl13 but not Arm mice; and **c-** This lethality was partially but significantly reversed by anti-PD1 treatment. These findings thus show that this mouse model generates consistent PD1^high^-expressing CTLs that do not effectively target a specific antigen for rejection. This is also validated by utilizing PD1 blockade to reverse the phenotype.

One advantage of this model is that tumor establishment occurs in the context of well-defined chronically exhausted T cells that are also found in tumor-infiltrating T-cells in brain (Figs 1 and 3). Systemic LCMV infection elicits a polyclonal GP33^+^ TCR T-cell response that matures from a naïve into an effector phenotype^39^. Therefore, T-cell responses after CT2A-gp33 tumor challenge would reflect the frequencies of pre-existent T cells that accumulate and expand in brains. Our model with LCMV-induced exhaustion in polyclonal T-cell pools against the antigens is comparable to the approach using adoptively transferred T-cells in recipient mice^14,40–42^. This is because specific antigen-specific T cells are traceable and these phenotypes can be manipulated in the donor mice. Despite this advantage in the adoptive T-cell transfer method, tumor-infiltrating lymphocytes appear with heterogeneous phenotypes and in different stages of maturation deriving from the adoptively transferred cells and recipient’s T-cell pool that respond to the same specific antigen^40,41^. In the model described in this paper, Cl13 infection-induced PD1^high^GP33^+^ CTL population become dysfunctional before encountering tumor and thus may more closely recapitulate the state of the immune system in advanced stages of cancer, such as recurrent GBM. One limitation of our model may be its mechanistic relevance since the process of antigen-specific T cell “exhaustion” occurs systemically in response to a viral infection and not locally in response to a “true” tumor antigen.

In naïve mouse models^18–22^, the effectiveness of PD-1 blockade is observed when the treatment starts early in tumor establishment (usually within the first two weeks of tumor-exposure), possibly because this treatment timing overlaps with the effector phase of primed CTL activation^2,43^. During this phase, these effector T cells are known to express PD-1 to modulate the magnitude of CTL proliferation via apoptosis and this early phase of PD-1 expression during CTL activation is thought to differ from the chronic expression of PD-1 that marks “exhausted” CTLs^2,30,33,42,44–47^. In this study, it appears that our model may mimic this ‘exhausted” CTL state even during the early stages of tumor establishment and thus allow for evaluation of immunotherapies (i.e. PD-1 blockade in here) in the presence of exhausted anti-tumor CTLs. Clinical trials of several immune checkpoint inhibitors are currently being pursued. A recent clinical trials using one PD-1 antibody for recurrent GBM patients was reported to show no response in recurrent GBM (NCT02017717) (D.A. Reardon, personal communication)^38^. Another clinical trial for newly diagnosed GBM patients after surgical resection is ongoing (NCT02667587). In our model, we only saw an effect of PD-1 blockade when it was delivered before and not after tumor challenge. This may imply that the current trials may be mostly effective when tumor burden is minimal, in agreement with other immunotherapy trials^48^.

When CTLs infiltrate in the tumor, several immunosuppressive events hinder effective tumor rejection. The tumor immunosuppressive microenvironment is composed of multiple factors, such as regulatory T cells, myeloid-derived suppressor cells, other innate immune cells and cytokines^13–15^. In fact, while we show the blockade of the PD-1 pathway did lead to GBM rejection (Fig. 4B), this protection was not absolute, indicating that effects of PD-1 blockade monotherapy may not completely restore the anti-tumor function of exhausted T cells, possibly because other immune checkpoint signals and epigenetic factors are also operative^2,4,5,33,44,49^. In addition, recent findings show that rescue by PD1-dependent therapies depends on an intact CD28 co-stimulatory pathway^50^. Furthermore, epigenetic programs have also been reported to exert a role in reversion of T cell fate^51^. Although T cells from Cl13-infected mice display the “exhausted” phenotype, it has been shown that they are still able to expand and mount a somewhat effective response, when adoptively transferred into naive mice who are then exposed to an acute LCMV infection^52^. However, we have not yet determined if PD1^*high*^ *GP33* ^+^ T cells from our mice would provide protection to naive mice challenged with CT2A-gp33 gliomas nor have we determined if there are significant differences in the memory compartments of Arm vs. Cl13 mice^51^. The role of these additional pathways in this model remain to be further explored.

We observed that PD-1^high^ GP33^+^ CTL in the brains of Cl13 mice (Fig. 3) could not reject tumor. Instead, Arm mice completely rejected the tumor, if PD-1^low^GP33^+^ CTLs accumulated in brains (Figs 2B and 3C **; CT2A vs CT2A-gp33**), even though they harbored a similar number of GP33-specific CTL to that of Cl13 mice in the PBMCs (Fig. 1A). To our knowledge, this is novel evidence linking “exhaustion” of CTL to GBM progression. The interaction between cancer and host is thought to lead to “cancer immunoediting,” where host immunity gradually shifts from a tumor rejection to a tumor escape role that includes impaired T-cell function and tumor antigen escape^24–26^. An example in GBM is the mutation (R132H) in the IDH1 gene, which can function as an immunogenic antigen initially recognized by CD4^+^ T cells, which then progressively lose their anti-tumor function^53,54^. Short-term (Arm) vs long-term (Cl13) exposure to the gp33 antigen may also skew the development of naïve T cells into memory T-cell vs. non-memory T cells (Fig. 1A). The observed downregulation of CD44 expression in Cl13 may also contribute to the “exhausted” state^55,56^ (supplementary Figure 1).

In conclusion, this experimental mouse model should be useful to further characterize the role of dysfunctional immunity in tumor progression.

## Materials and Methods

### DNA constructs

The immunodominant LCMV epitope GP33-41 was synthesized (IDT, Coralville, Iowa) and cloned in pENTR/D-TOPO (Life Technologies). Inserted sequences were confirmed by DNA sequencing (Eton Bioscience, Boston MA). The fragments inserted between attL1 and attL2 sites were further transferred into the plenti-PGK-puro-DEST vector (addgene, Cambridge MA) using Gateway LR Clonase II kit (Life Technologies). Packaging of lentiviral vectors was conducted in 293FT cells (Life Technologies) by transfecting these constructs with pMD2.G and psPAX2 packaging vectors as described previously^57^.

### Cell lines

Mouse CT-2A glioma cells were initially generated by Dr. Tomas Seyfried (Boston College, Boston MA) and tested as negative for Mycoplasma infection in the Laboratory of Virology and Epidemiology of Yale University^58^. They were cultured in Dulbecco’s Modified Eagle Medium (D-MEM) supplemented with 10% fetal bovine serum (FBS) and 10 mM HEPES (4-(2-hydroxyethyl)-1-piperazineethanesulfonic acid) buffer. All media and supplements were obtained from Thermo Fisher Scientific (Waltham, MA). Cells were infected with the lentiviral vector that encodes the human PGK promoter-driving the LCMV epitope peptide sequences, GP33-41 (GP33: M-KAVYNFATM). The resulting infected cells were diluted and selected using 5 µg/mL puromycin (Sigma) to isolate clonal cell populations that were then analyzed by RT-PCR (data not shown) and T cell proliferation assays to identify the GP33 epitope expressing clones (Fig. 1C). The primers and probes in this study were designed by PrimerQuest Tool (IDT; Coralville, Iowa) as follows: 18 s rRNA (VIC-AGTTGGTGGAGCGATTTGTCTGGT-QSY, 5′-CACGGACAGGATTGACAGATT-3′ and 5′-GCCAGAGTCTCGTTCGTTATC-3′), WPRE (6-FAM-TGCTGACGC/ZEN/AACCCCCACTGGT-IBFQ, 5′-CCGTTGTCAGGCAACGTG-3′ and 5′-AGCTGACAGGTGGTGGCAAT-3′), GP33 (5′-AATTTCGCCACCATGTGAAAG-3′ and 5′-GGGCCACAACTCCTCATAAA-3′). For the T-cell proliferation assay, splenocytes obtained from mice, pre-infected with Arm, were co-incubated with RT-PCR positive clones for 5 hours and subsequently analyzed by fluorescence-activated cell sorting (FACS), as described in Fig. 1C. One of the clones, CTgp33.7 (clone 7), was selected for this study and named CT2A-gp33. After establishing this cell line, expanded cells were cryopreserved to minimize passages (no more than ten) before implanting *in vivo*.

### *In vivo* animal studies

*All e*xperimental procedures using animals were carried out under an animal protocol reviewed and approved by the Harvard Center for Comparative Medicine (HCCM) and BWH’s IACUCs, and performed in accordance with relevant guidelines and regulations. C56Bl/6 J mice were purchased from The Jackson Laboratories (Bar Harbor, ME) and infected with LCMV Armstrong by intraperitoneal injection with 2 × 10^5^ pfu or LCMV clone-13 by intravenous injection with 4 × 10^6^ pfu. At 3-weeks after LCMV infection, CT-2A cells (1 × 10^5^ in 5 uL Hank’s Balanced Salt Solution or HBSS) and CT2A-gp33 (4 × 10^5^ cells) were injected intracranially at stereotactic coordinates (ventral 3.5, Rostral 0.5 and right lateral 2.0 (in mm) from bregma) using a stereotaxic apparatus (David Korp Instruments; Tujunga, CA). For antibody treatment, PD-1 Ab (29 F.1A12) or isotype control IgG (2A3) were obtained from BioXcell (West Lebanon, NH), diluted in phosphate-buffered saline (PBS) and administered at doses of 10 mg per kg body weight by intraperitoneal injection. Brain tissues were collected after perfusion with chilled PBS buffer and dissociated with enzyme mixture in RPMI1640 with 5% FBS at the following doses: 30 U/ml of DNase I type IV, 0.1 mg/ml of Hyaluronidase type V, and 1 mg/ml of Collagenase type IV (all from Sigma). After washing dissociated cells with RPMI with 2% FBS, lymphocytes were enriched in the 67–44% Percoll gradient solution (GE Healthcare) at 2,000 rpm for 20 minutes at room temperature. Peripheral blood mononuclear cells (PBMCs) were enriched using Ficoll-Plaque Plus solution (GE Healthcare) at 1,900 rpm for 20 minutes. Single-cell suspensions from spleens were prepared by passing cells through 70-µm cell strainers and Ammonium-Chloride-Potassium (ACK) lysis.

### Antibodies and flow cytometry

For surface staining, fluorophore-conjugated mAbs specific for CD3 (17A2), CD44 (IM7), CD279/PD-1 (RMP1-30), CD127 (A7R34), CD62L (MEL-14), H-2D[b] (KH95), I-A[b] (AF6-120.1) were obtained from Biolegend (San Diego, CA), CD45(30-F11), CD274/PD-L1 (MIH5), CD8a (53–6.7) were from BD Biosciences (San Jose, CA). Live/Dead Near-IR Dead cell stain kit, and APC-streptavidin were from Thermo Fisher Scientific (Waltham, MA). For GP33 tetramer staining, the biotinylated class I monomer, obtained from the National Institutes of Health Tetramer Core Facility (Emory University, GA), was conjugated with APC-streptavidin to form the tetramer. For intracellular IFNγ staining, 1 × 10^7^ splenocytes (Figs 1C and 3A) or total number of Percoll-isolated lymphocytes (Fig. 3D) were stimulated at 37 °C for 5 h with 1 × 10^6^ tumor cells or 1 µg/ml GP33-41 peptides (GenScript) in the presence of GolgiStop and GolgiPlug (BD). The cells were washed and surface stained before intracellular staining with anti-IFNγ (XMG1.2) using BD Cytofix/Cytoperm™ Plus solution kit. Cells were run on an LSR II (BD Biosciences) at CCVR Flow Cytometry Core in Beth Israel Deaconess Medical Center (BIDMC; Boston, MA), and analyses were performed with FlowJo (TreeStar).

### Statistical analysis

All analyses were completed using the Prism 6 (GraphPad). Unadjusted pairwise comparison p-value for the log-rank test was used for survival analyses (Figs 1C and 3B), and Wilcoxon rank-sum test p-values for comparing treatments or conditions (Figs 2C,D and 3C–E). Kaplan-Meier survival plots and whiskers in box plots were generated with Prism 6 and formatted with Illustrator CS6 (Adobe) Software.

## Electronic supplementary material

Supplementary figure 1
