# Supplementary material for: Modeling tumor immunity of mouse glioblastoma by exhausted CD8+ T cells
Source: Sci Rep. 2018 Jan 9;8:208. doi: 10.1038/s41598-017-18540-2 (PMC5760520; doi:10.1038/s41598-017-18540-2)
Supplement: Supplementary file 1 — Supplementary figure 1 [file 41598_2017_18540_MOESM1_ESM.doc]

**Supplementary data for:**

**Modeling tumor immunity of mouse glioblastoma by exhausted CD8^+^ T cells.**

**Hiroshi Nakashima^1*^, Quazim A. Alayo^1^, Pablo Penaloza-MacMaster^2^, Gordon J. Freeman^3^, Vijay K. Kuchroo^4^, David A. Reardon^5^, Soledad Fernandez^6^, Michael Caligiuri^7^, and E. A. Chiocca^1*^**

^1^Harvey W. Cushing Neuro-oncology Laboratories (HCNL), Department of Neurosurgery, Harvard Medical School and Brigham and Women’s Hospital, Boston, MA 02115, US

^2^Department of Microbiology-Immunology, Feinberg School of Medicine, Northwestern University, Chicago, IL 60611

^3^Department of Medical Oncology, Dana-Farber Cancer Institute, and Brigham and Women’s Hospital, Boston, MA 02115

^4^Evergrande Center for Immunologic Diseases, Harvard Medical School and Brigham and Women’s Hospital, Boston, MA 02115

^5^Center for Neuro-Oncology, Dana-Farber Cancer Institute, and Brigham and Women’s Hospital, Boston, MA 02115

^6^Center for Biostatistics, The Ohio State University, Columbus, Ohio, USA 43210

^7^Comprehensive Cancer Center, and Division of Hematology in Department of Internal Medicine, College of Medicine, The Ohio State University, Columbus, Ohio, USA 43210

*Corresponding Authors: E. A. Chiocca, Address, 75 Francis Street, Boston, MA 02115; Phone, 617-732-6939; Fax, 1-617-734-8342; Email, [EAChiocca@bwh.harvard.edu](mailto:EAChiocca@bwh.harvard.edu)

Hiroshi Nakashima, Address 60 Fenwood Road #8016, Boston, MA 02115; Phone, 617-525-5058; Fax, 617-525-8698; Email, [HNakashima@bwh.harvard.edu](mailto:HNakashima@bwh.harvard.edu)

**Conflict of Interest statement**

GF has patents/pending royalties on the PD-1 pathway. Other authors declare no potential conflicts of interest.

**Running title:** Novel glioblastoma model to examine tumor rejection

Figure S1


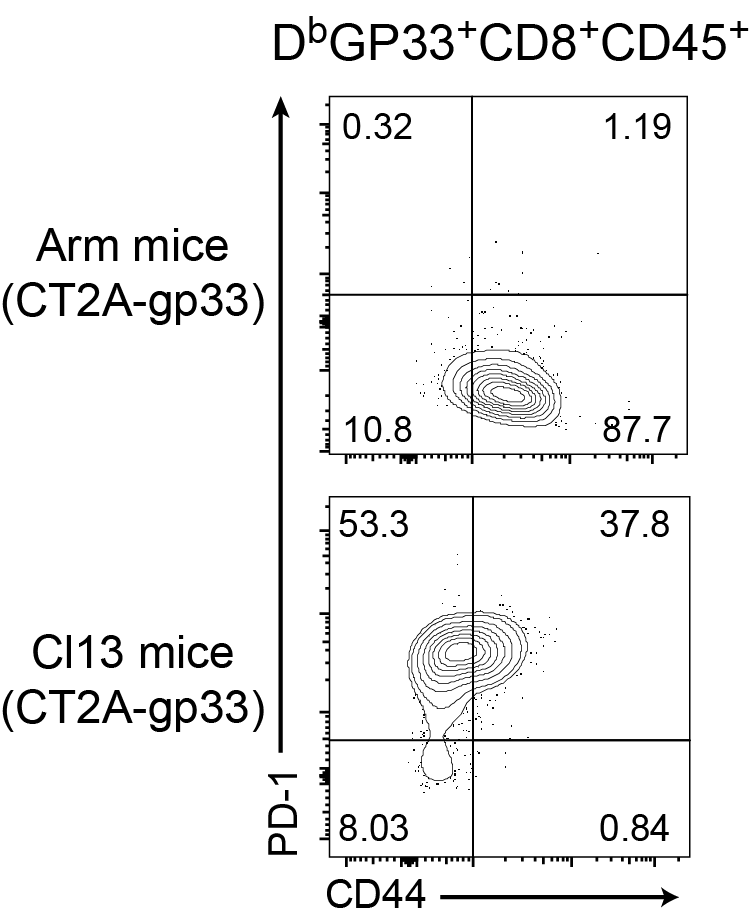


A. FACS plot of brain-infiltrating lymphocytes from the same data of Arm and Cl13 infected mice shown in Fig 3B. PD-1 and CD44 expression are shown in the plot after gating on Live+CD45+CD8+D^b^GP33+ T cells.
